# Radiation combined with temozolomide contraindicated for young adults diagnosed with anaplastic glioma

**DOI:** 10.18632/oncotarget.11756

**Published:** 2016-08-31

**Authors:** Pei Yang, Chuanbao Zhang, Jinquan Cai, Gan You, Yinyan Wang, Xiaoguang Qiu, Shouwei Li, Chenxing Wu, Kun Yao, Wenbin Li, Xiaoxia Peng, Wei Zhang, Tao Jiang

**Affiliations:** ^1^ Department of Neurosurgery, Beijing Tiantan Hospital, Capital Medical University, Beijing, China; ^2^ Beijing Neurosurgical Institute, Capital Medical University, Beijing, China; ^3^ Department of Radiation Therapy, Beijing Tiantan Hospital, Capital Medical University, Beijing, China; ^4^ China National Clinical Research Center for Neurological Diseases, Beijing, China; ^5^ Department of Neurosurgery, Beijing Sanbo Brain Hospital, Capital Medical University, Beijing, China; ^6^ Department of Pathology, Beijing Sanbo Brain Hospital, Capital Medical University, Beijing, China; ^7^ Department of Oncology, Beijing Shijitan Hospital, Capital Medical University, Beijing, China; ^8^ Center of Brain Tumor, Beijing Institute for Brain Disorders, Beijing, China; ^9^ Department of Neurosurgery, The Second Affiliated Hospital of Harbin Medical University, Harbin, China; ^10^ Department of Epidemiology and Biostatistics, School of Public Health and Family Medicine, Capital Medical University, Beijing, China

**Keywords:** high-grade glioma, young adult, radiation, temozolomide, survival

## Abstract

**Purpose:**

Age is a major prognostic factor for malignant gliomas. However, few studies have investigated the management of gliomas in young adults. We determined the role of survival and treatment in young adults with advanced gliomas in a large population from the Chinese Glioma Genome Atlas (CGGA).

**Methods:**

This study included 726 adults (age ≥ 18) with histologically proven anaplastic glioma or glioblastoma multiforme (GBM). The overall and progression-free survival was determined in young (age < 50) and older groups (age ≥ 50).

**Results:**

The study included an older group (OP) of 264 patients and a younger group (YP) of 462patients. In the OP group with GBM and anaplastic glioma, patients treated with RT combined with temozolomide (TMZ) manifested significantly longer OS and PFS compared with patients assigned to RT alone (P < 0.05). In contrast, the YP group diagnosed with anaplastic glioma failed to show any survival advantage with RT plus TMZ compared with RT alone.

**Conclusions:**

We observed no survival benefit in young adults (age < 50) with anaplastic glioma when treated with TMZ combined with RT. Our findings warrant further investigation of younger patients diagnosed with anaplastic glioma treated with radiotherapy plus TMZ chemotherapy.

## INTRODUCTION

Malignant gliomas rank among the most prevalent primary intracranial neoplasms in adults [1], with an incidence of 80%. [2] Based on the the World Health Organization (WHO) criteria [3], grade IV glioblastoma multiforme (GBM) accounts for almost 65% of all the gliomas. The average survival is poor and age-dependent.[4] Anaplastic glioma (grade III) is a diverse group of malignancies comprising anaplastic astrocytoma (AA), anaplastic oligoastrocytoma (AOA) and anaplastic oligodendroglioma (AO). It is less frequently diagnosed and is associated with better prognosis compared with grade IV glioblastoma, despite shared molecular features and poor outcomes in the elderly.[5, 6]

Surgical resection or biopsy, and involved-field radiotherapy are indicated for the treatment of glioblastomas or anaplastic gliomas. Radiation and chemotherapy with temozolomide (TMZ) is the standard of care for patients with glioblastomas.[7]

The prognosis of grade II - IV malignant glioma is largely dependent on age. Recent studies have mainly focused on older patients and suggest that the benefit of treatment is reduced with age. [8] Cranial irradiation is associated with an increased risk of cognitive impairment.[9] Further, older patients are poorly tolerant to radiotherapy combined with TMZ.[10] Other studies suggest that older patients with a good performance status benefit from radiotherapy [11] and possibly from chemotherapy.[12] In addition, the ANOCEF Phase II results also indicated that TMZ was safe in elderly patients with GBM and poor KPS.[13] Conversely, few studies have focused on the treatment and survival of younger adults (age < 50 years).

In this study, we summarized the clinical management and evaluated the role of age in clinical outcomes of patients diagnosed with grade III and IV gliomas. We determined the clinical efficacy of treatment across different ages, especially younger adults with advanced gliomas in a large population in the Chinese Glioma Genome Atlas (CGGA).

## RESULTS

### Patients

In this study, we analyzed 726 patients diagnosed with advanced (WHO grade III and IV) gliomas from the Chinese Glioma Genome Atlas (CGGA).

Patient demographics are listed in Table 1.The study population included a higher number of males (61%) than females. The study included 264 older patients (OP) and 462 younger patients (YP). The median age of OP was 58 years (ranging from 50 to 83). The median age of YP was 39 years (ranging from 18-49). Sixty percent of all patients had a preoperative KPS ≥ 80, including 53% in OP and 64% in YP, respectively. The histopathological diagnosis of patients treated surgically was established by two neuropathologists according to the 2016 WHO classification system. Grade III anaplastic glioma in 81 patients (31%) of the OP group, included 20 patients (8%) with anaplastic astrocytoma, 16 (6%) with anaplastic oligodendroglioma, and 45 (17%) with oligoastrocytoma. Grade IV GBM was found in 183 patients of the OP (69%) group. In YP, Grade III anaplastic glioma was detected in 201 patients (44%), including 55 (12%) with anaplastic astrocytoma, 35 (8%) with anaplastic oligodendroglioma, and 111 (24%) with oligoastrocytoma. Grade IV GBM was found in 261 patients in YP (56%).

**Table 1:** Baseline patient characteristics

		Total (*n*=726,%)	Age≥50 (*n*=264, 36%)	Age<50 (*n*=462, 64%)	*P* value
Age	Median (range)	45 (18-83)	58 (50-83)	39 (18-49)	
Gender	Male	443 (61)	161 (61)	282 (61)	P=0.9885
	Female	283 (39)	103 (39)	180 (39)	
Presenting symptoms	Increased ICP	294 (40)	110 (42)	184 (40)	*P*<0.0001
	Epilepsy	129 (18)	31 (12)	98 (21)	
	Neurologic deficit	204 (28)	105 (40)	99 (21)	
KPS score	Preoperative KPS≥80	437 (60)	140 (53)	297 (64)	*P*=0.0029
	Preoperative KPS<80	289 (40)	124 (47)	165 (36)	
Pathological type	AA	75 (10)	20 (8)	55 (12)	*P*=0.0076
	AO	51 (7)	16 (6)	35 (8)	
	AOA	156 (21)	45 (17)	111 (24)	
	GBM	444 (61)	183 (69)	261 (56)	
Side of tumor	Left	334 (46)	119 (45)	215 (47)	*P*=0.8060
	Right	333 (46)	125 (47)	208 (45)	
	Bilateral	59 (8)	20 (8)	39 (8)	
Tumor location (involved)	Frontal lobe	333 (46)	102 (39)	231 (50)	*P*=0.0294
	Temporal lobe	264 (36)	103 (39)	161 (35)	
	Parietal lobe	114 (16)	47 (18)	67 (15)	
	Occipital lobe	74 (10)	35 (13)	39 (8)	
	Insular lobe	80 (11)	27 (10)	53 (11)	
Resection	Gross total resection	424 (58)	154 (58)	270 (58)	*P*=0.9773
	Subtotal	302 (42)	110 (42)	192 (4)	
Postoperative Treatment	RT plus TMZ	352 (48)	131 (50)	221 (48)	*P*=0.7597
	RT	90 (12)	36 (14)	54 (12)	
	TMZ	38 (5)	11 (4)	27 (6)	
	Supportive Treatment	68 (9)	25 (9)	43 (9)	
	NA	178 (25)	61 (23)	117 (25)	
IDH1 mutation	Mutation	184 (25)	34 (13)	150 (32)	*P*<0.0001
	Wildtype	470 (65)	205 (78)	265 (57)	
	NA	72 (10)	25 (9)	47 (10)	
1p LOH	Deletion	43 (6)	13 (5)	30 (6)	*P*=0.6526
	No deletion	430 (59)	156 (59)	274 (59)	
	NA	253 (35)	95 (36)	158 (34)	
19q LOH	Deletion	46 (6)	12 (5)	34 (7)	*P*=0.3193
	No deletion	427 (59)	157 (59)	270 (58)	
	NA	253 (35)	95 (36)	158 (34)	
1p/19q codeletion	Deletion	36 (5)	8 (3)	28 (6)	*P*= 0.1913
	No deletion	437 (60)	161 (61)	276 (60)	
	NA	253 (35)	95 (36)	158 (34)	
MGMT promoter methylation	Methylated	236 (32)	88 (33)	148 (32)	*P*<0.0001
	Not methylated	229 (32)	105 (40)	124 (27)	
	NA	261 (36)	71 (27)	190 (41)	

The standard treatment for malignant gliomas consists of surgery, postoperative radiotherapy, combined with adjuvant TMZ chemotherapy. Surgical resection is the first choice. Gross total resection was conducted in 424 patients (58 %) including 154 (58%) OP and 270 (58%) YP cases, respectively. In the OP group, 131 (50 %) patients were treated with postoperative radiotherapy, and TMZ chemotherapy (RT+TMZ), 36 (14%) underwent postoperative radiotherapy alone (RT), 11 (4%) received postoperative TMZ chemotherapy alone (TMZ) and 25 (9%) were managed with supportive treatment. In the YP group, 221 (48%) were treated with postoperative radiotherapy and TMZ chemotherapy (RT+TMZ), 54 (12%) received postoperative radiotherapy alone (RT), 27 (6%) received postoperative TMZ chemotherapy alone (RT) and 43 (9%) were managed with supportive treatment (Table 1).

### Genetic aberrations

Patients with adequate tumor specimens were analyzed for genetic changes including isocitrate dehydrogenase 1 (*IDH1*) mutations, 1p/19q loss of heterozygosity (LOH) and promoter methylation of O6-methylguanine-DNA methyltransferase (MGMT). The number and frequency of alterations in each age group are listed in Table 1. In the OP group, 35 tumors (13 %) harbored *IDH1* mutations, 34 tumors (5 %) carried LOH on 1p, 12 cases (5 %) showed LOH on 19q, 8 cases (3 %) exhibited 1p/19q co-deletion and 88 tumors (33 %) revealed *MGMT* promoter methylation. In the YP group, *IDH1* mutations were detected in 150 cases (32 %), much higher than in OP (P < 0.0001). The other genetic alterations in the YP group were as follows: LOH involving 1p in 30 cases (6 %), LOH involving 19q in 34 cases (7 %), 1p/19q co-deletions in 28 cases (6 %) and MGMT promoter methylation in 148 tumors (32 %).

### Survival

**Figure 1:**
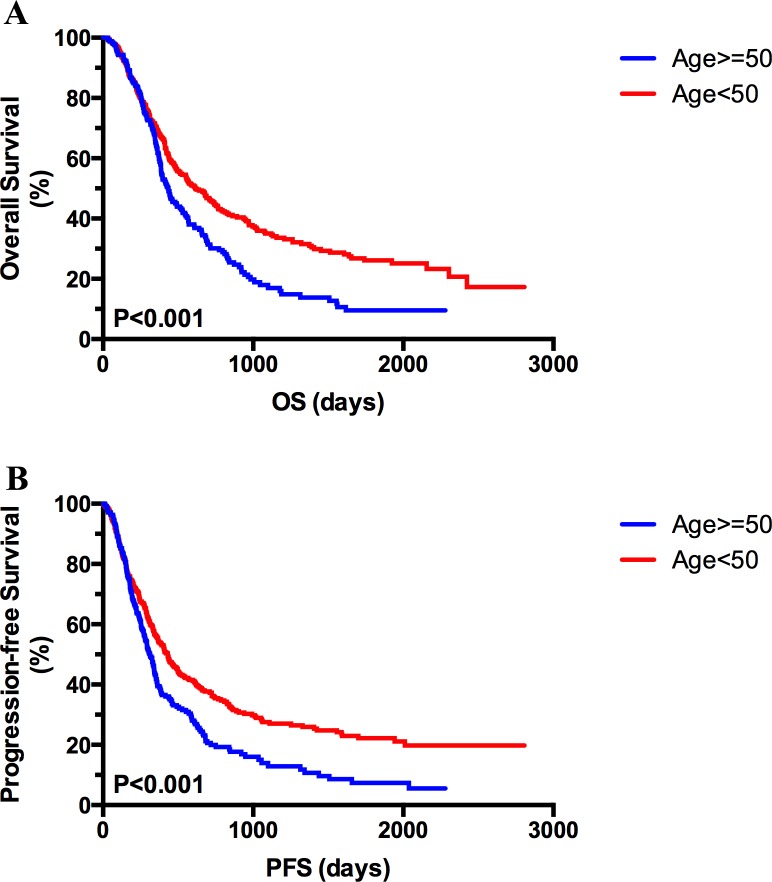
Kaplan–Meier analysis of overall survival (OS) and progression-free survival (PFS) based on age Kaplan–Meier curves showed better prognosis in patients aged < 50 compared with older patients (age ≥ 50) in terms of OS (**A**) and PFS (**B**)

The 243 patients with anaplastic glioma (86%) and 406 patients with GBM (91%) were followed up. Among the two age-specific subgroups with advanced gliomas, the prognosis of YP was more favorable than in OP in terms of overall survival (OS) and progression-free survival (PFS) (P < 0.001; Figure 1A and 1B). The median OS of patients in the OP group treated with RT plus TMZ, RT alone and supportive treatment was 17.9, 12.9 and 10.5 months, respectively. In the YP group, the median OS of patients treated with RT combined with TMZ, RT alone and supportive treatment was 25.8, 21.3 and 11.1 months, respectively, which were significantly longer than in the OP group. In the OP group, the median PFS with RT plus TMZ, RT alone and supportive treatment was 12.3, 8.4 and 9.3 months, respectively. In the YP group, the median PFS of the above three groups was 17.3, 13.6 and 6.0 months, respectively. The OS and PFS of OP and YP groups exposed to different treatments are listed in Table 2.

**Table 2:** First-line therapy of patients according to age stratification

	Patients with Age ≥ 50			Patients with Age < 50		
	RT + TMZ	RT alone	Supportive treatment	RT + TMZ	RT alone	Supportive treatment
No. of patients	131	36	25	221	54	43
Median OS (month)	17.9	12.9	10.5	25.8	21.3	11.1
at 6-month (%)	92	86	68	92	73	65
at 1-year (%)	62	48	41	79	59	40
at 3-year (%)	29	15	7	37	41	17
at 5-year (%)	19	8	NA	28	33	11
Median PFS (month)	12.3	8.4	9.3	17.3	13.6	6
at 6-month (%)	77	51	59	81	64	50
at 1-year (%)	48	27	32	62	50	29
at 3-year (%)	20	12	7	30	34	11
at 5-year (%)	17	8	NA	25	29	NA

### Survival correlated with age, tumor grade and therapeutic strategy

**Figure 2:**
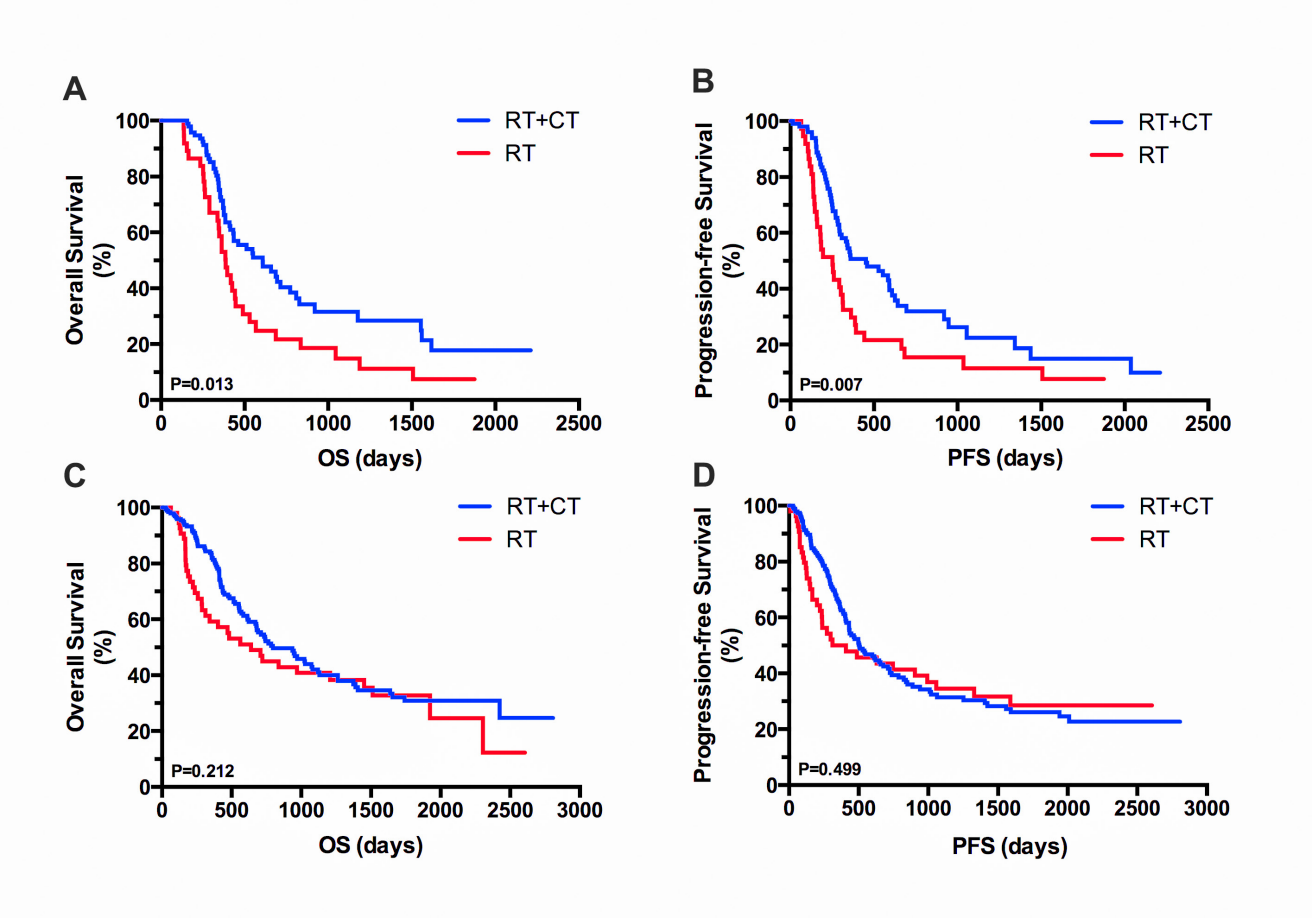
Kaplan–Meier analysis of OS and PFS based on treatment in OP and YP groups Kaplan–Meier curves show significant differences in the OP group in both OS (**A**) and PFS (**B**) following treatment with RT plus TMZ and RT alone. By contrast, in the YP group, no significant differences in OS (**C**) or PFS (**D**) were observed with RT plus TMZ.

Kaplan–Meier analysis of OS and PFS in the different age groups is presented in Figure 2. In the OP, both OS and PFS were significantly longer in the RT plus TMZ group than in RT group (P = 0.013, Figure 2A and P = 0.007, Figure 2B). By contrast, no significant benefit in OS and PFS was observed in the YP group managed with RT plus TMZ chemotherapy (P = 0.212, Figure 2C and P = 0.499, Figure 2D).

**Figure 3:**
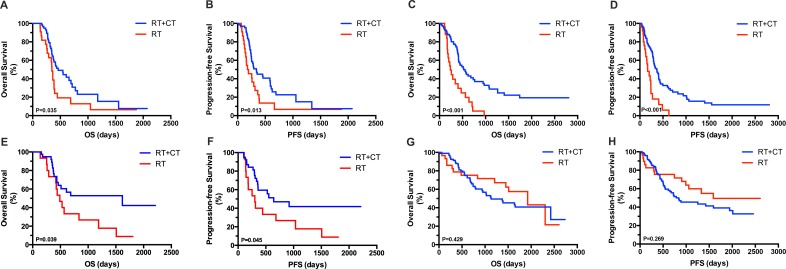
Kaplan–Meier analysis of OS and PFS according to tumor grade and treatment assignment in OP and YP groups OP patients with GBM treated postoperatively with RT plus TMZ showed improved OS (**A**) and PFS (**B**). YP patients with GBM assigned to RT plus TMZ also showed significantly longer OS (**C**) and PFS (**D**) compared with those treated with RT alone. OP patients with anaplastic gliomas assigned to RT plus TMZ exhibited significantly longer OS (**E**) and PFS (**F**) compared with patients treated with RT alone. By contrast, in YP patients with WHO grade III gliomas, treatment with RT plus TMZ yielded no survival benefit when compared with treatment using RT alone, in terms of both OS (**G**) and PFS (**H**).

In the OP group of patients with GBM, a favorable OS and PFS was observed among patients treated postoperatively with RT plus TMZ (P = 0.035, Figure 3A and P = 0.013, Figure 3B). In the YP group diagnosed with GBM, patients assigned to RT and TMZ exhibited significantly longer OS and PFS compared with those administered RT alone (P < 0.001, Figure 3C and P < 0.001, Figure 3D).

In the OP group diagnosed with anaplastic gliomas (WHO grade III), patients treated with RT plus TMZ showed significantly longer OS and PFS compared with those treated with RT alone (P = 0.039, Figure 3E and P=0.045, Figure 3F). By contrast, no survival advantage was found in the YP group diagnosed with WHO grade III gliomas when treated with RT plus TMZ compared with RT alone in terms of OS (P = 0.429, Figure 3G) and PFS (P = 0.269, Figure 3H).

### Survival correlated with genetic mutations and therapeutic strategies

To determine the correlation between patient survival, and genetic alterations and therapeutic strategies, we analyzed *IDH1* mutations, 1p/19q co-deletions and *MGMT* promoter methylation. Table 3 summarizes the genetic changes in each subgroup (age < 50 or ≥ 50, RT+TMZ or RT alone). No significant differences were found among the different treatment groups in terms of genetic alterations in both anaplastic gliomas and GBMs. In all the patients in the OP and YP groups diagnosed with GBMs, the optimal survival benefit was always found among patients treated with RT combined with TMZ regardless of MGMT promoter methylation. In the YP group with anaplastic gliomas, no survival benefit was observed with RT plus TMZ compared with RT alone, independent of genetic alterations. We also performed Kaplan-Meier analysis of OS and PFS based on genetic changes in the different age groups (Supplemental Figure 1).

**Table 3:** Comparison of genetic alternation between different subgroups according to age and treatment

N (%)	Anaplastic glioma						GBM					
	OP		*P*	YP		*P*	OP		*P*	YP		*P*
	RT+TMZ	RT alone		RT+TMZ	RT alone		RT+TMZ	RT alone		RT+TMZ	RT alone	
mIDH	11 (20)	4 (7)	0.91	42 (32)	20 (15)	0.0855	7 (7)	1 (1)	0.5642	22 (18)	4 (3)	0.8755
wtIDH	28 (52)	11 (20)		55 (42)	13 (10)		75 (73)	20 (19)		80 (66)	16 (13)	
methMGMT	23 (44)	9 (17)	0.8846	63 (54)	19 (16)	0.3489	20 (23)	9 (10)	0.2232	33 (32)	5 (5)	0.2743
unmethMGMT	14 (27)	6 (12)		24 (21)	11 (9)		46 (53)	11 (13)		50 (49)	14 (14)	
codele1p/19q	3 (8)	2 (5)	0.5587	14 (14)	3 (3)	0.3021						
uncodele1p/19q	24 (63)	9 (24)		56 (58)	24 (25)							

### Multivariate analysis

**Figure 4:**
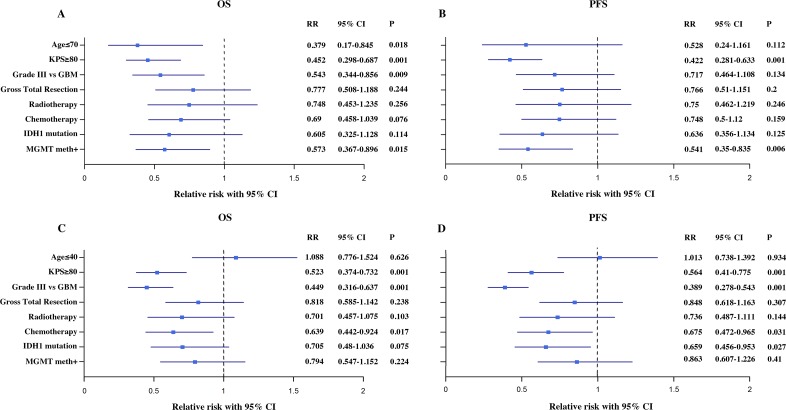
Cox regression analysis of variables related to OS and PFS in OP (**A** and **B**) and YP (**C** and **D**) Cox model for age (age ≤ 70 vs. > 70 for OP and age ≤ 40 vs. > 40 for YP), KPS ≥ 80 vs. < 80, Grade III vs. GBM, gross total resection vs. no gross total resection, radiotherapy vs. no radiotherapy, TMZ chemotherapy vs. no TMZ chemotherapy, *IDH1* mutation vs. no *IDH1* mutation, *MGMT* promoter methylation vs. no *MGMT* promoter methylation.

Multivariate analysis of OS and PFS included age, KPS, tumor grade, extent of resection, radiotherapy, TMZ chemotherapy, IDH1 mutation and *MGMT* promoter methylation (Figure 4).

In the OP group with advanced gliomas, age ≤ 70 (P = 0.018), higher KPS score (KPS ≥ 80, P < 0.001) and MGMT promoter methylation (P = 0.015) were significantly associated with OS in multivariate Cox analysis (Figure 4A). A higher KPS (KPS ≥ 80, P < 0.001) and gene methylation (P = 0.006) were significantly associated with PFS (Figure 4B). In the YP group diagnosed with advanced gliomas, higher KPS (KPS ≥ 80, P < 0.001), lower WHO grade (grade III, P < 0.001) and TMZ chemotherapy (P = 0.017) were independent risk factors for OS (Figure 4C). The prognostic risk factors for PFS included higher KPS (KPS ≥ 80, P < 0.001), lower WHO grade (grade III, P < 0.001), TMZ chemotherapy (P = 0.031) and *IDH1* mutation (P = 0.027) (Figure 4D).

## DISCUSSION

Advances in radiotherapy [14, 15] and treatment with nitrosourea-based interventions led to limited therapeutic success.[16] Temozolomide, [17] first used for the treatment of recurrent malignant gliomas, appeared promising for recurrent WHO grade III gliomas. However, the effective response of glioblastomas ranged between 5% and 8%.[18, 19] The role of TMZ chemotherapy was undefined [20] until the EORTC 26981 trial was published. The trial found a significantly prolonged survival in GBM patients treated with adjuvant TMZ chemotherapy.[7]

Since 2005, patients diagnosed with WHO grade III and IV gliomas were recommended TMZ combined with RT. However, patients are unable to afford TMZ therapy due to lack of insurance coverage.[21] We, therefore, analyzed patients exposed to the combination therapyand RT alone. We found a higher efficacy of TMZ in older patients than in younger patients.In the current study, we validated previous evidence suggesting that the combination of RT and TMZ enhanced the survival of GBM patients compared with RT alone, despite age stratification.[7] In contrast, younger patients with anaplastic gliomas (WHO grade III) manifested greater survival following treatment with RT combined with TMZ compared with RT alone.

Age is an independent prognostic factor in grade II-IV malignant glioma. Compared with aged and elderly patients, younger adults show a relatively better prognosis as well as improved general condition. They also exhibit greater tolerance to surgical resection. Anaplastic glioma or glioblastoma patients carrying mutant *IDH1* or *IDH2* are significantly younger than those harboring wild-type *IDH1* and *IDH2*.[22, 23] Our findings are consistent with these studies in that the frequency of *IDH* mutations was low in patients aged above 50 (13% vs. 32%, P < 0.0001). The NOA-04 trial and validation cohorts in NOA-08 and the German Glioma Network indicated that methylation of *MGMT* promoter improved outcomes in patients carrying wild-type *IDH*.[24] However, the present study offered no definitive evidence suggesting that *IDH* mutations increased the benefit of adjuvant TMZ chemotherapy. A comparison of genetic alterations across different subgroups according to age and treatment, yielded no significant differences in anaplastic gliomas and GBMs. This finding suggests that age was one of the prognostic risk factors in patients treated with adjuvant TMZ chemotherapy for anaplastic glioma.

Age, extent of resection and tumor grade are established prognostic factors for gliomas. According to clinical practice guidelines [25, 26], both tumor grade and extent of resection are primary risk factors for therapeutic decision-making. However, it remains unclear whether age is an independent prognostic factor in malignant gliomas, especially in younger adults. In this study, we mainly focused on younger adults diagnosed with high-grade gliomas, in an effort to correlate survival with treatment in age-specific subgroups. In spite of the limitations of this retrospective study, we demonstrated no survival benefit in younger adults aged under 50, with anaplastic gliomas treated with RT combined with TMZ compared with RT alone. Conversely, older adults with anaplastic gliomas and GBMs may benefit from RT combined with TMZ treatment. Furthermore, the specific genetic alterations were not prognostic indicators for TMZ chemotherapy in any patients.

Our findings may trigger discussion involving younger adults diagnosed with anaplastic glioma in the combination therapy arm. The positive outcome indicated that RT or TMZ alone was adequate for younger patients diagnosed with anaplastic glioma, which warrants further validation in prospective randomized studies

## MATERIALS AND METHODS

### Patients' clinical demographics

Adult patients aged at least 18 years and diagnosed with advanced gliomas (WHO III-IV: AA, AO, AOA and GBM) in the Chinese Glioma Genome Atlas (CGGA) were retrospectively studied. All patients were managed surgically, followed by postoperative radiotherapy, and concomitant and/or adjuvant TMZ chemotherapy at the Glioma Treatment Center of Beijing Tiantan Hospital and Beijing Sanbo Brain Hospital, from October 2004 to July 2012. The study was approved by the hospital ethics committees. All the patients provided written informed consent. The histological diagnosis was validated by two independent neuropathologists and graded according to the 2016 WHO criteria.[3] Patients' clinical records were reviewed for age at diagnosis, sex, presenting symptoms, preoperative Karnofsky performance status (KPS) score, and surgical status. Patients at least 50 years of age were defined as Older Patients (OP) while those under 50 years were categorized as Younger Patients (YP). Overall survival (OS) was defined as the period starting from operation until death. OS data were primarily collected during patients' visit to the clinic and via phone interviews with patients and their relatives. Progression-free survival (PFS) was recorded starting with surgery until radiographic progression. The exclusion criteria were: patients lost to follow-up or death from secondary diseases.

### Treatment

Standard care comprised surgery, postoperative adjuvant radiotherapy, and concomitant and adjuvant TMZ chemotherapy. The primary goal of surgery was maximal tumor bulk resection excluding the cortex. MRI findings were used to determine tumor characteristics and the extent of resection within 48 h post-surgery. Abnormal preoperative fluid-attenuated inversion recovery (FLAIR) signals were used to compare the extent of resection (gross or subtotal) based on neuroradiologist reports.[27] The extent of resection was independently determined by two experienced radiologists, who were blinded to the clinical data. The initial postoperative interventions were classified into postoperative radiotherapy combined with concomitant and adjuvant TMZ chemotherapy (RT/TMZ→TMZ) or postoperative radiotherapy alone (RT) for patients with GBMs; postoperative radiotherapy in addition to adjuvant TMZ chemotherapy (RT→TMZ), adjuvant TMZ chemotherapy alone (TMZ) or postoperative radiotherapy alone (RT) for patients with anaplastic gliomas. Routine postoperative adjuvant radiotherapy was administered to patients within four weeks after surgery. The total dose of 54-60 Gy was administered over 30 days, in daily doses of 1.8-2 Gy, and 5 fractions were administered each week. Concomitant chemotherapy comprised a daily dose of TMZ (75 mg/m2) over seven days weekly starting with the first until the last day of radiotherapy, for a maximum of 49 days. After a four-week hiatus, patients were administered up to six cycles of adjuvant oral TMZ (150–200 mg/m2) for 5 days every 28 days. The chemotherapy regimen included a total of 6 cycles in the absence of disease progression or irreversible hematological toxicity.

### Molecular evaluation

Tumor tissue samples were resected surgically before starting radio- or chemotherapy. The tissue specimens were snap-frozen and stored in liquid nitrogen until further use.

Genomic DNA was isolated using the QIAamp DNA Mini Kit (Qiagen). The DNA samples were analyzed using the Nano-Drop ND-1000 spectrophotometer (NanoDrop Technologies, Houston, TX). The sample specimems were analyzed for *IDH1* mutations (R132 site, DNA pyro-sequencing), *MGMT* promoter methylation (DNA pyro-sequencing) and 1p/19q co-deletion (fluorescence *in situ* hybridization).[28, 29]

### Survival and follow-up

Survival was monitored clinically during patient visits and via telephone interviews. Patients who were biopsied were excluded from this study. The baseline examination included magnetic resonance imaging (MRI), total blood counts, hematological tests, and physical examinations. During radiotherapy (with or without TMZ), patients were monitored weekly. Comprehensive investigations included physical and radiological assessments 21 to 28 days after radiotherapy and every 3 months subsequently. Adjuvant TMZ therapy included monthly clinical evaluation and comprehensive assessment toward the end of the third and sixth cycles. Tumor progression was defined by a 25% increase in tumor size, new lesions, or an increased need for corticosteroid therapy.[30]

### Statistical analyses

SPSS 13.0 software (USA) was used to analyze the data. The differences in clinicopathological characteristics between older and younger adult patients were evaluated with X2 test. OS and PFS were estimated using Kaplan–Meier analysis and compared with two-sided log-rank test. Cox (proportional-hazards) regression analysis was used to assess the prognostic role of the clinicopathological factors and statistically significant treatment protocols based on univariate testing. A P-value < 0.05 was considered statistically significant.

## SUPPLEMENTARY MATERIAL



## References

[1] Ostrom Q.T, Gittleman H, Fulop J, Liu M, Blanda R, Kromer C, Wolinsky Y, Kruchko C, Barnholtz-Sloan J.S (2015). CBTRUS Statistical Report: Primary Brain and Central Nervous System Tumors Diagnosed in the United States in 2008-2012. Neuro Oncol.

[2] Ricard D, Idbaih A, Ducray F, Lahutte M, Hoang-Xuan K, Delattre J.Y (2012). Primary brain tumours in adults. Lancet.

[3] Louis D.N, Ohgaki H, Wiestler O.D, Cavenee W.K, Burger P.C, Jouvet A, Scheithauer B.W, Kleihues P (2007). The 2007 WHO classification of tumours of the central nervous system. Acta Neuropathol.

[4] Ostrom Q.T, Gittleman H, Farah P, Ondracek A, Chen Y, Wolinsky Y, Stroup N.E, Kruchko C, Barnholtz-Sloan J.S (2013). CBTRUS statistical report: Primary brain and central nervous system tumors diagnosed in the United States in 2006-2010. Neuro Oncol.

[5] Barnholtz-Sloan J.S, Williams V.L, Maldonado J.L, Shahani D, Stockwell H.G, Chamberlain M, Sloan A.E (2008). Patterns of care and outcomes among elderly individuals with primary malignant astrocytoma. J Neurosurg.

[6] Hartmann C, Hentschel B, Wick W, Capper D, Felsberg J, Simon M, Westphal M, Schackert G, Meyermann R, Pietsch T, Reifenberger G, Weller M, Loeffler M (2010). Patients with IDH1 wild type anaplastic astrocytomas exhibit worse prognosis than IDH1-mutated glioblastomas, and IDH1 mutation status accounts for the unfavorable prognostic effect of higher age: implications for classification of gliomas. Acta Neuropathol.

[7] Stupp R, Mason W.P, van den Bent M.J, Weller M, Fisher B, Taphoorn M.J, Belanger K, Brandes A.A, Marosi C, Bogdahn U, Curschmann J, Janzer R.C, Ludwin S.K (2005). Radiotherapy plus concomitant and adjuvant temozolomide for glioblastoma. N Engl J Med.

[8] Stupp R, Hegi M.E, Mason W.P, van den Bent M.J, Taphoorn M.J, Janzer R.C, Ludwin S.K, Allgeier A, Fisher B, Belanger K, Hau P, Brandes A.A, Gijtenbeek J (2009). Effects of radiotherapy with concomitant and adjuvant temozolomide versus radiotherapy alone on survival in glioblastoma in a randomised phase III study: 5-year analysis of the EORTC-NCIC trial. Lancet Oncol.

[9] Weller M, Platten M, Roth P, Wick W (2011). Geriatric neuro-oncology: from mythology to biology. Curr Opin Neurol.

[10] Sijben A.E, McIntyre J.B, Roldan G.B, Easaw J.C, Yan E, Forsyth P.A, Parney I.F, Magliocco A.M, Bernsen H, Cairncross J.G (2008). Toxicity from chemoradiotherapy in older patients with glioblastoma multiforme. J Neurooncol.

[11] Keime-Guibert F, Chinot O, Taillandier L, Cartalat-Carel S, Frenay M, Kantor G, Guillamo J.S, Jadaud E, Colin P, Bondiau P.Y, Menei P, Loiseau H, Bernier V (2007). Radiotherapy for glioblastoma in the elderly. N Engl J Med.

[12] Glantz M, Chamberlain M, Liu Q, Litofsky N.S, Recht L.D (2003). Temozolomide as an alternative to irradiation for elderly patients with newly diagnosed malignant gliomas. Cancer.

[13] Gallego Perez-Larraya J, Ducray F, Chinot O, Catry-Thomas I, Taillandier L, Guillamo J.S, Campello C, Monjour A, Cartalat-Carel S, Barrie M, Huchet A, Beauchesne P, Matta M (2011). Temozolomide in elderly patients with newly diagnosed glioblastoma and poor performance status: an ANOCEF phase II trial. J Clin Oncol.

[14] Laperriere N, Zuraw L, Cairncross G (2002). Cancer Care Ontario Practice Guidelines Initiative Neuro-Oncology Disease Site. Radiotherapy for newly diagnosed malignant glioma in adults: a systematic review. Radiother Oncol.

[15] Miralbell R, Mornex F, Greiner R, Bolla M, Storme G, Hulshof M, Bernier J, Denekamp J, Rojas A.M, Pierart M, van Glabbeke M, Mirimanoff R.O (1999). Accelerated radiotherapy, carbogen, and nicotinamide in glioblastoma multiforme: report of European Organization for Research and Treatment of Cancer trial 22933. J Clin Oncol.

[16] Stewart L.A (2002). Chemotherapy in adult high-grade glioma: a systematic review and meta-analysis of individual patient data from 12 randomised trials. Lancet.

[17] Newlands E.S, Stevens M.F, Wedge S.R, Wheelhouse R.T, Brock C (1997). Temozolomide: a review of its discovery, chemical properties, pre-clinical development and clinical trials. Cancer Treat Rev.

[18] Yung W.K, Albright R.E, Olson J, Fredericks R, Fink K, Prados M.D, Brada M, Spence A, Hohl R.J, Shapiro W, Glantz M, Greenberg H, Selker R.G (2000). A phase II study of temozolomide vs. procarbazine in patients with glioblastoma multiforme at first relapse. Br J Cancer.

[19] Brada M, Hoang-Xuan K, Rampling R, Dietrich P.Y, Dirix L.Y, Macdonald D, Heimans J.J, Zonnenberg B.A, Bravo-Marques J.M, Henriksson R, Stupp R, Yue N, Bruner J (2001). Multicenter phase II trial of temozolomide in patients with glioblastoma multiforme at first relapse. Ann Oncol.

[20] Grossman S.A, O'Neill A, Grunnet M, Mehta M, Pearlman J.L, Wagner H, Gilbert M, Newton H.B, Hellman R, G. Eastern Cooperative Oncology (2003). Phase III study comparing three cycles of infusional carmustine and cisplatin followed by radiation therapy with radiation therapy and concurrent carmustine in patients with newly diagnosed supratentorial glioblastoma multiforme: Eastern Cooperative Oncology Group Trial 2394. J Clin Oncol.

[21] Yang P, Zhang W, Wang Y, Peng X, Chen B, Qiu X, Li G, Li S, Wu C, Yao K, Li W, Yan W, Li J (2015). IDH mutation and MGMT promoter methylation in glioblastoma: Results of a prospective registry. Oncotarget.

[22] Yan H, Parsons D.W, Jin G, McLendon R, Rasheed B.A, Yuan W, Kos I, Batinic-Haberle I, Jones S, Riggins G.J, Friedman H, Friedman A, Reardon D (2009). IDH1 and IDH2 mutations in gliomas. N Engl J Med.

[23] Zhang C.B, Bao Z.S, Wang H.J, Yan W, Liu Y.W, Li M.Y, Zhang W, Chen L, Jiang T (2014). Correlation of IDH1/2 mutation with clinicopathologic factors and prognosis in anaplastic gliomas: a report of 203 patients from China. J Cancer Res Clin Oncol.

[24] Wick W, Meisner C, Hentschel B, Platten M, Schilling A, Wiestler B, Sabel M.C, Koeppen S, Ketter R, Weiler M, Tabatabai G, von Deimling A, Gramatzki D (2013). Prognostic or predictive value of MGMT promoter methylation in gliomas depends on IDH1 mutation. Neurology.

[25] Brem S.S, Bierman P.J, Black P, Blumenthal D.T, Brem H, Chamberlain M.C, Chiocca E.A, DeAngelis L.M, Fenstermaker R.A, Fine H.A, Friedman A, Glass J, Grossman S.A (2005). Central nervous system cancers: Clinical Practice Guidelines in Oncology. J Natl Compr Canc Netw.

[26] Jiang T, Mao Y, Ma W, Mao Q, You Y, Yang X, Jiang C, Kang C, Li X, Chen L, Qiu X, Wang W, Li W (2016). CGCG clinical practice guidelines for the management of adult diffuse gliomas. Cancer Lett.

[27] McGirt M.J, Chaichana K.L, Attenello F.J, Weingart J.D, Than K, Burger P.C, Olivi A, Brem H, Quinones-Hinojosa A (2008). Extent of surgical resection is independently associated with survival in patients with hemispheric infiltrating low-grade gliomas. Neurosurgery.

[28] Yan W, Zhang W, You G, Bao Z, Wang Y, Liu Y, Kang C, You Y, Wang L, Jiang T (2012). Correlation of IDH1 mutation with clinicopathologic factors and prognosis in primary glioblastoma: a report of 118 patients from China. PLoS One.

[29] Wick W, Hartmann C, Engel C, Stoffels M, Felsberg J, Stockhammer F, Sabel M.C, Koeppen S, Ketter R, Meyermann R, Rapp M, Meisner C, Kortmann R.D (2009). NOA-04 randomized phase III trial of sequential radiochemotherapy of anaplastic glioma with procarbazine, lomustine, and vincristine or temozolomide. J Clin Oncol.

[30] Macdonald D.R, Cascino T.L, Schold S.C., Cairncross J.G (1990). Response criteria for phase II studies of supratentorial malignant glioma. J Clin Oncol.

