# Supplementary material for: Radiation combined with temozolomide contraindicated for young adults diagnosed with anaplastic glioma
Source: Oncotarget. 2016 Aug 31;7(48):80091–100. doi: 10.18632/oncotarget.11756 (PMC5346774; doi:10.18632/oncotarget.11756)
Supplement: Supplementary file 1 [file oncotarget-07-80091-s001.pdf]

Radiation combined with temozolomide contraindicated for young adults diagnosed with anaplastic glioma

Supplementary Material

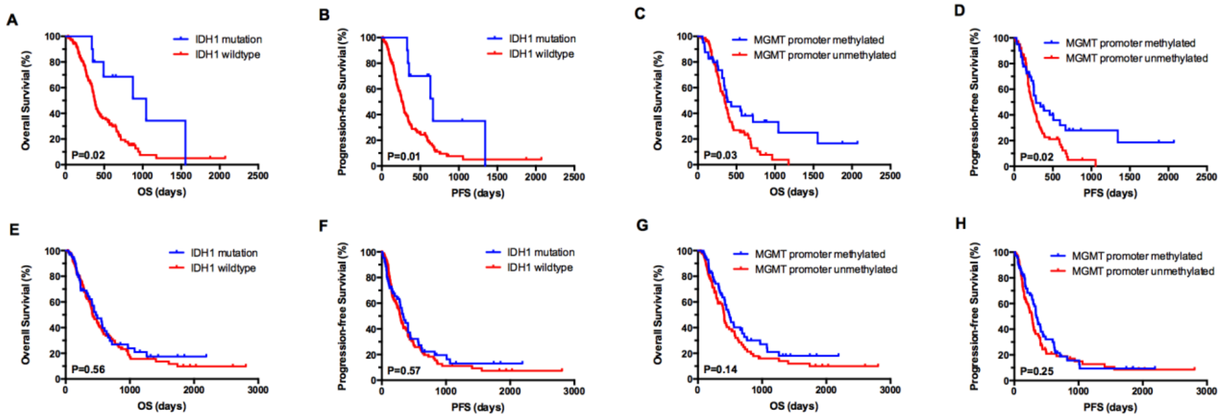

Supplemental Fig. 1 Kaplan–Meier analysis of OS and PFS in the presence of *IDH1* mutations and *MGMT* promoter methylation in OP and YP.

Kaplan-Meier analysis of OS and PFS in patients with and without *IDH1* mutation and *MGMT* promoter methylation in OP (A, B, C and D) and YP (E, F, G and H).
